# Biochemical and histological evidence of deteriorated bioprosthetic valve leaflets: the accumulation of fibrinogen and plasminogen

**DOI:** 10.1242/bio.034009

**Published:** 2018-08-15

**Authors:** Tomohisa Sakaue, Hirotomo Nakaoka, Fumiaki Shikata, Jun Aono, Mie Kurata, Teruyoshi Uetani, Mika Hamaguchi, Ai Kojima, Shunji Uchita, Takumi Yasugi, Haruhiko Higashi, Jun Suzuki, Shuntaro Ikeda, Jitsuo Higaki, Shigeki Higashiyama, Hironori Izutani

**Affiliations:** 1 Department of Cardiovascular and Thoracic Surgery, Ehime University Graduate School of Medicine, Ehime 791-0295, Japan; 2 Department of Cell Growth and Tumor Regulation, Proteo-Science Center (PROS), Toon, Ehime 791-0295, Japan; 3 Division of Laboratory Animal Research, Advanced Research Support Center (ADRES), Toon, Ehime 791-0295, Japan; 4 Department of Cardiothoracic Surgery, St Vincent's Hospital Sydney, NSW 791-0295, Australia; 5 Department of Cardiology, Pulmonology, Hypertension, and Nephrology, Ehime University Graduate School of Medicine, Ehime 791-0295, Japan; 6 Department of Pathology, Division of Analytical Pathology, Ehime University Graduate School of Medicine, Ehime 791-0295, Japan; 7 Department of Pathology, Proteo-Science Center (PROS), Toon, Ehime 791-0295, Japan; 8 Department of Biochemistry and Molecular Genetics, Ehime University Graduate School of Medicine, Toon, Ehime 791-0295, Japan

**Keywords:** Aortic valve, Bioprosthetic valve, Calcification, Fibrinogen, Plasminogen

## Abstract

Calcification of bioprosthetic valves (BVs) implanted in aortic position can result in gradual deterioration and necessitate aortic valve replacement. The molecular mechanism of calcium deposition on BV leaflets has been investigated, but remains to be fully elucidated. The present study aimed to identify explanted bioprosthetic valve (eBV)-specific proteins using a proteomics approach and to unveil their biochemical and histological involvements in calcium deposition on BV leaflets. Calcification, fibrosis, and glycosylation of the valves were histologically assessed using Von Kossa, Masson's Trichrome and Alcian Blue staining, as well as immunostaining. Protein expression in the explanted biological valves was analysed using proteomics and western blotting. In a histological evaluation, αSMA-positive myofibroblasts were not observed in eBV, whereas severe fibrosis occurred around calcified areas. SDS-PAGE revealed three major bands with considerably increased intensity in BV leaflets that were identified as plasminogen and fibrinogen gamma chain (100 kDa), and fibrinogen beta chain (50 and 37 kDa) by mass analysis. Immunohistochemistry showed that fibrinogen β-chain was distributed throughout the valve tissue. On the contrary, plasminogen was strongly stained in CD68-positive macrophages, as evidenced by immunofluorescence. The results suggest that two important blood coagulation-related proteins, plasminogen and fibrinogen, might affect the progression of BV degeneration.

## Abstract (precis)

**Summary:** Fibrinogen was specifically deposited on whole deteriorated tissue valve leaflets, and plasminogen-positive macrophages strongly invaded the areas around calcified bioprosthetic and native tissues.

## INTRODUCTION

Aortic valve stenosis (AS), defined as thickened and calcified leaflets with decreased mobility, is the most prevalent valvular heart disorder (Alame et al., 2017). The main causes of AS are heart disorders, such as rheumatic disease and bicuspid aortic valve and the degeneration of valves resulting from atherosclerosis (Mol et al., 2009). At present, early surgical or trans-catheter aortic valve replacement (AVR) is the only therapeutic choice for severe AS.

Bioprosthetic valve (BV) leaflets have been preferably used for AVR in patients over 60 years of age (Alame et al., 2017) because mechanical valves are associated with major complications, including bleeding related to life-long anti-coagulation therapy and thromboembolisms (Basude et al., 2012; Nishimura et al., 2017). The BV leaflets are pre-treated with glutaraldehyde to avoid immunologic reactions and calcium deposition. However, implanted BV leaflets can deteriorate because of structural disruption by calcification and fibrin thrombus a decade and a half after implantation (Basude et al., 2012; Simionescu et al., 2012). The molecular mechanisms of calcification and fibrosis in the valves have not been unveiled. Therefore, basic and clinical studies are ongoing to identify the determinants of calcification and fibrosis occurrence and progression in implanted BVs.

In this study, we successfully obtained deteriorated BV leaflets from patient undergoing redo aortic valve replacement and characterised biochemical and histological features. We found that fibrinogen and plasminogen were specifically localised in explanted (e)BV leaflets in aortic position. Here, we first report the histological differences between eBV and native valves.

## RESULTS

### Histological characterisation of valve leaflets from patients with AS

**Fig. 1.**
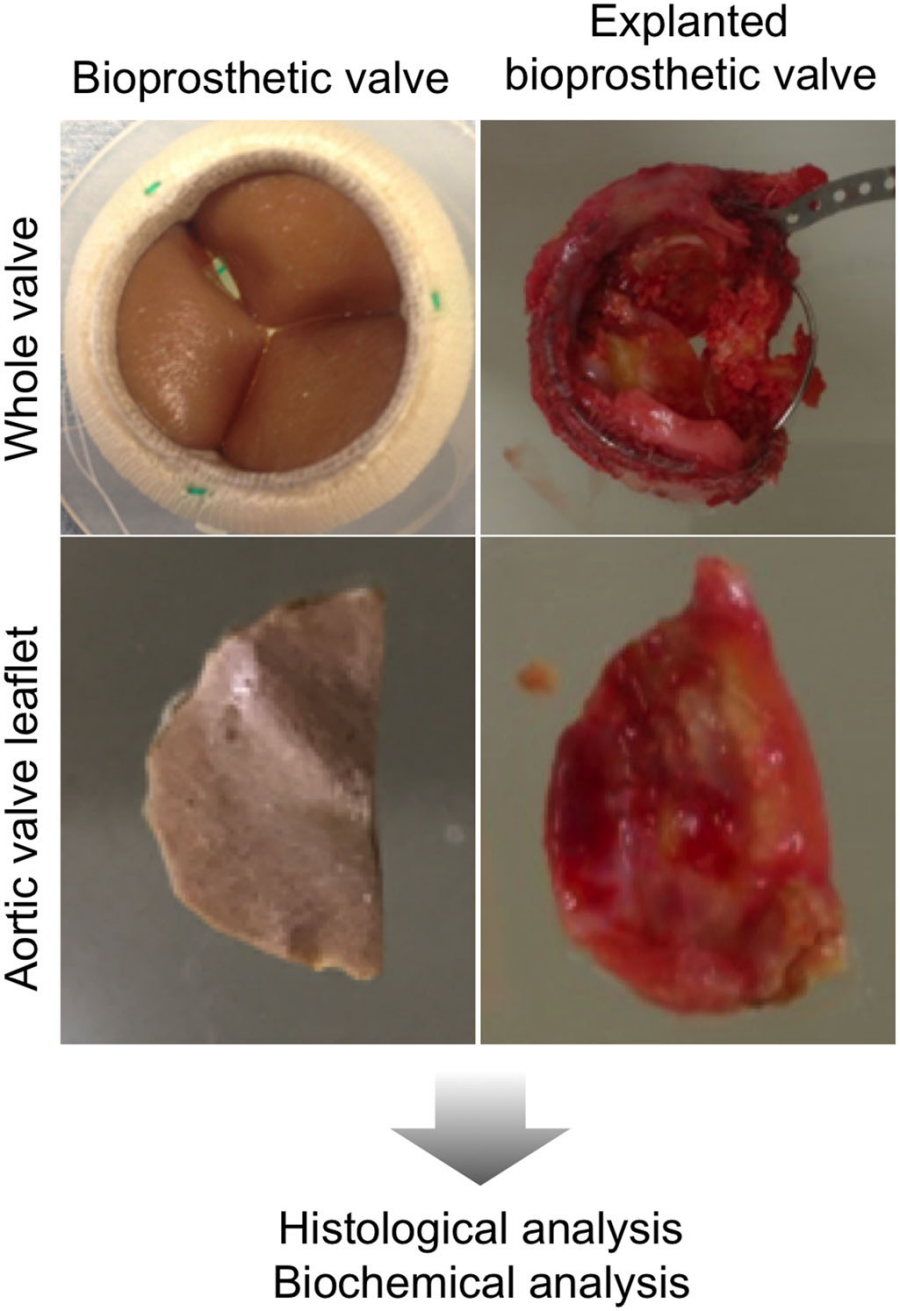
**Experimental strategy for biochemical and histological analyses of explanted and pre-implanted bioprosthetic valves.** Whole view (upper panels) and leaflet view (lower panels) of explanted (left panels) and pre-implanted (right panels) aortic BV leaflets.

**Fig. 2.**
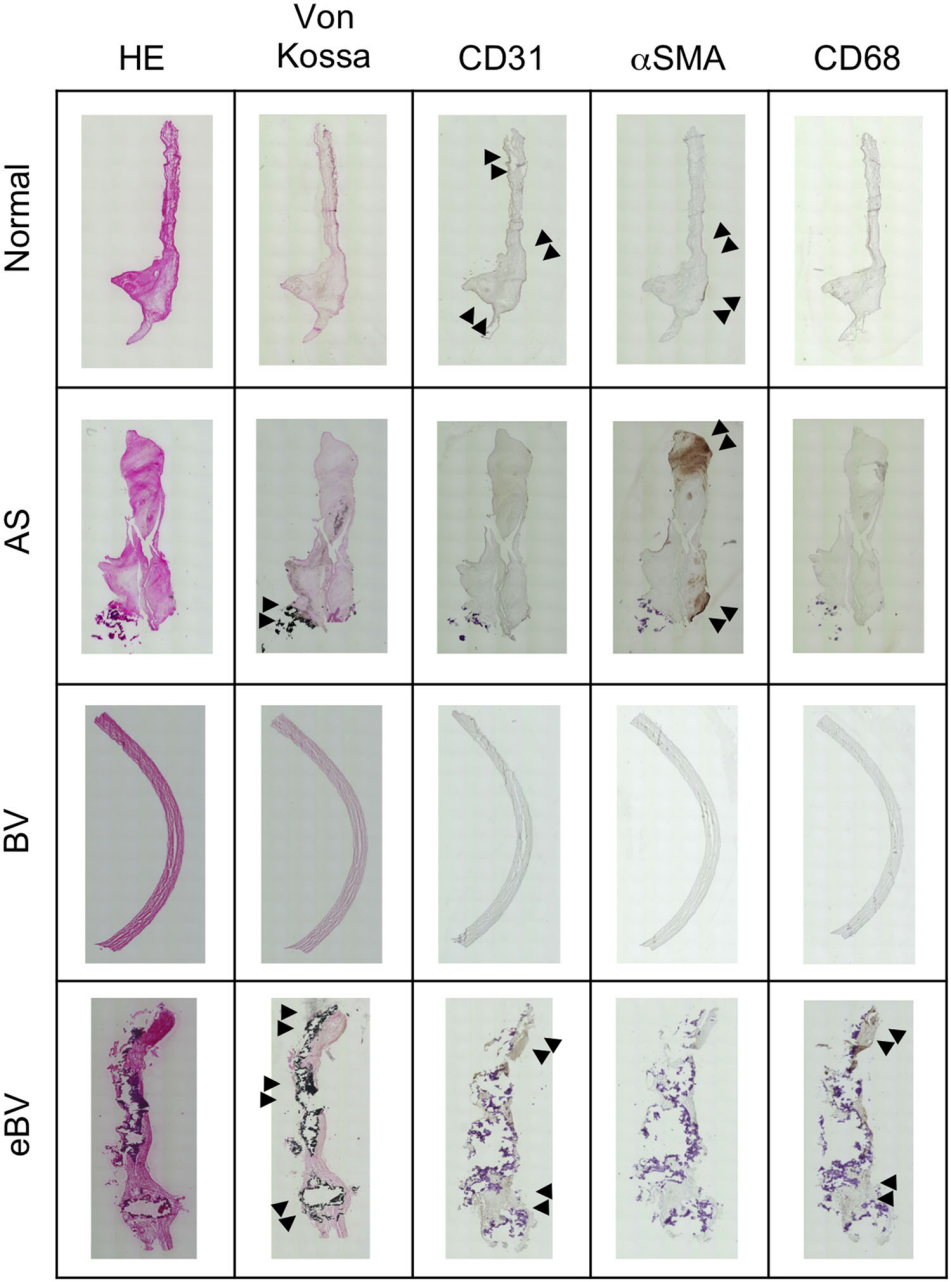
**Histological analyses of various aortic valve leaflets.** Valve leaflets were dissected from normal valve, AS valve, aortic eBV and unused aortic BV. The leaflets were stained with H&E and Von Kossa and immunostained for CD31, αSMA and CD68. Arrowheads indicate stained areas. All images were taken with a BZ-9000 instrument (Keyence) and displayed as tiled images.

To clarify the pathological features of calcified BV leaflets, we obtained eBVs from AVR patients and unused commercially available Magna EASE aortic valves (Edwards Lifesciences LLC, Irvine, CA) (Fig. 1). Valve leaflets were dissected out from the eBVs and histologically analysed by immunostaining for CD31 (endothelial cells), αSMA (myofibroblasts), and CD68 (macrophages), and by Von Kossa staining for calcium deposition, in comparison with valves collected from necropsied patients. To observe entire valve leaflets, we took pictures using the tiling function of BZ-9000 (Keyence, Osaka, Japan). AS valves and eBV leaflets were strongly hypertrophied as compared to Magna EASE control BV and normal valves (Fig. 2). In addition, AS valves and eBV leaflets were strongly stained with Von Kossa stain. Valvular endothelial cells were aligned on both the aortic and the ventricular side of normal valves and eBV leaflets. CD68-positive macrophages were dominantly located around calcified areas in AS and eBVs (Fig. 2). Interestingly, in contrast to these similarities between AS and eBVs, the localisation of αSMA-positive myofibroblasts was substantially different; while they were widely distributed in stromal tissues of AS valves, only few or no αSMA-positive myofibroblasts were observed in those of eBV (Fig. 2). These phenotypes were seen in all valve leaflets. Contrarily, pre-implant Magna EASE was not stained by any of the staining procedures used in this study. These results suggested that there might be large differences in the molecular mechanism of calcification progression between AS and eBVs (Fig. 2).

**Fig. 3.**
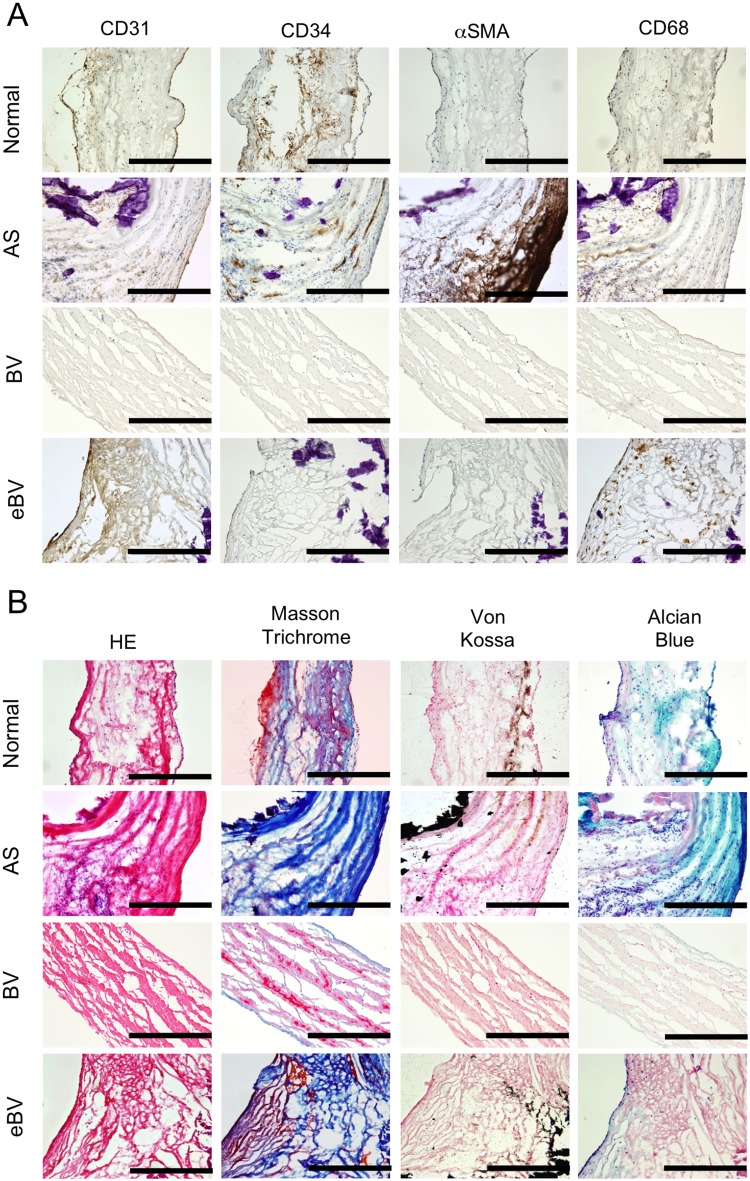
**Combination analyses of special stains and immunostains of aortic valve tissues.** (A) Immunohistological staining for CD31, CD34, αSMA and CD68 and (B) histological analyses by H&E, Masson's Trichrome, Von Kossa and Alcian Blue of leaflets from normal, AS, BV and eBV. Scale bars: 100 μm.

Based on our findings regarding the localisation of αSMA-positive cells, we conducted additional histological analyses, including Masson's Trichrome and Alcian Blue staining, to investigate the fibrosis and glycosylation of valve leaflets in detail. Invaded CD68-positive macrophages, CD34-positive stromal cells, and αSMA-positive myofibroblasts were clearly observed around the calcified tissues in the valves of AS patients (Fig. 3A), while these stained cells were completely abrogated by absence of primary antibodies (data not shown). Masson's Trichrome staining revealed that fibrosis was well developed in AS valves and eBVs. Whereas AS and normal valves were strongly stained by Alcian Blue (Fig. 3B), used to determine aortic valve area, eBVs and BV leaflets were not. On the contrary, Masson's Trichrome staining revealed strong fibrosis in calcified BV leaflets, although no myofibroblasts were observed in the tissue (Fig. 3B). These results suggested that myofibroblasts might not be essential for fibrosis and calcification in eBVs.

### Proteomic analyses of calcified BV leaflets

**Fig. 4.**
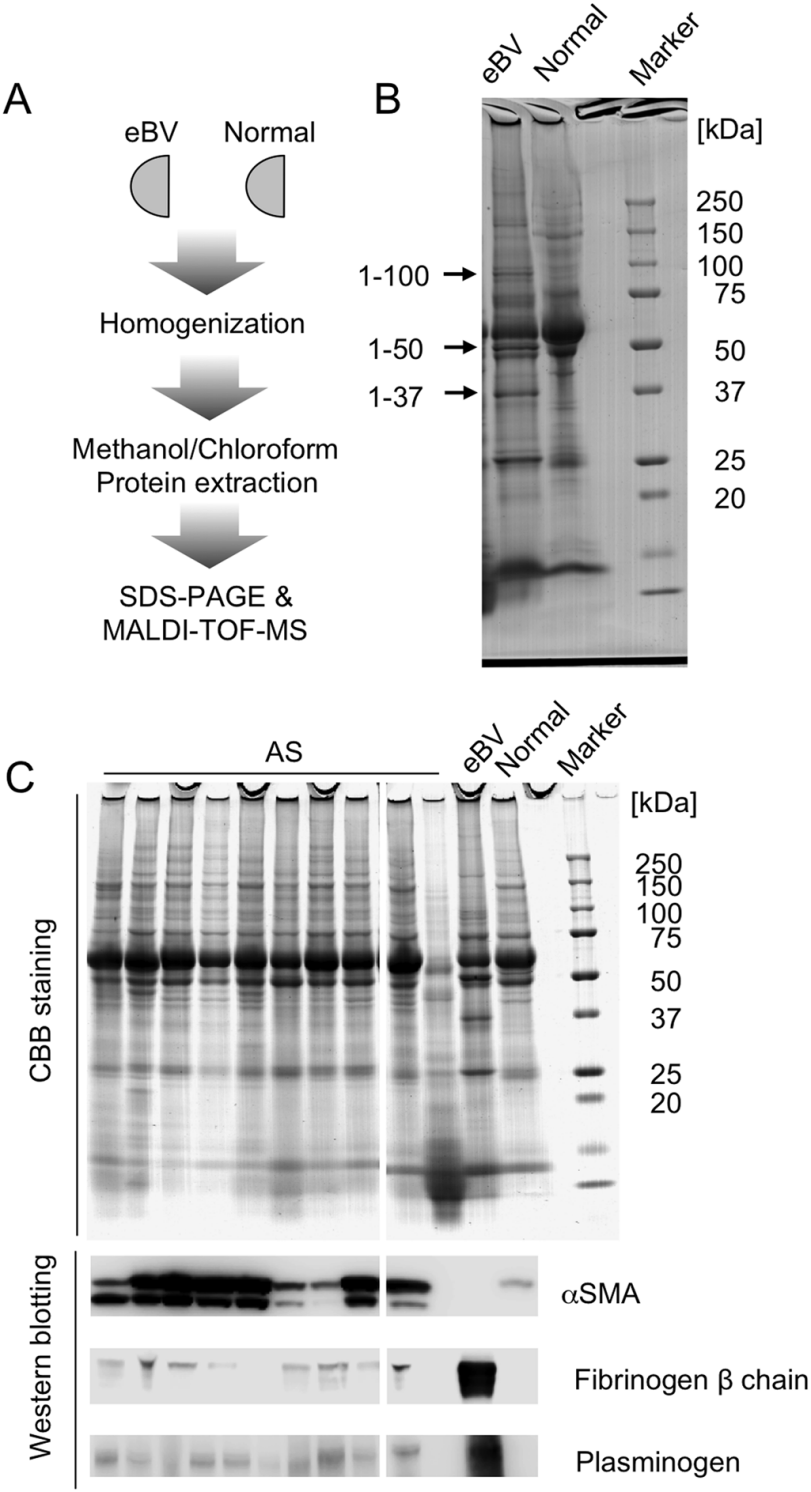
**Molecular screening of aortic bioprosthetic valve deterioration using proteomics.** (A) Experimental strategy for protein profiling of tissue lysates from eBV and normal valves. (B) SDS-PAGE of tissue lysates from eBV and normal valves. The three bands indicated with an arrow were analysed by MALDI-TOF-MS. (C) CBB staining (upper panels) and western blotting (lower panels) for αSMA, fibrinogen beta chain and plasminogen of valve tissue lysates from AS, eBV and normal valves.

To identify eBV tissue-specific proteins, we carried out sodium dodecyl sulphate-polyacrylamide gel electrophoresis (SDS-PAGE) and matrix-assisted laser desorption ionisation time-of-flight coupled with mass spectrometry (MALDI-TOF-MS) of proteins extracted from eBVs and normal leaflets (Fig. 4A). As shown in Fig. 4B and Table 1 three proteins were more highly expressed in eBVs than in normal valves. These proteins were identified as plasminogen and fibrinogen gamma chain (100 kDa), and fibrinogen beta chain (50 and 37 kDa) by MALDI-TOF-MS based on their molecular masses (Table 1). Subsequently, to investigate the expression levels of these proteins in eBVs, 20 μg of extracted crude proteins from normal and calcified aortic valves and eBVs were subjected to SDS-PAGE (Fig. 4C), followed by western blotting for plasminogen, fibrinogen beta chain, and αSMA. As shown in Fig. 4C, αSMA levels were dramatically increased in AS patients, while this protein was not detected in tissue lysates from eBVs and normal valves. These results were in accordance with the immunostaining results (Figs 2 and 3). Fibrinogen beta chain was clearly detected in eBV leaflets, but was hardly detected in lysates from normal and AS valves (Fig. 4C). On the contrary, plasminogen was highly expressed in eBVs as well as AS valves, but not in normal valves. These findings suggested that these three proteins might be strongly associated with the pathological processes of calcification and fibrosis in BV leaflets.

**Table 1.**
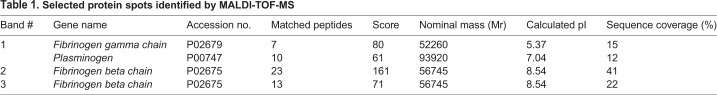
**Selected protein spots identified by MALDI-TOF-MS**

### Localisation of fibrinogen and plasminogen in various valve tissues

**Fig. 5.**
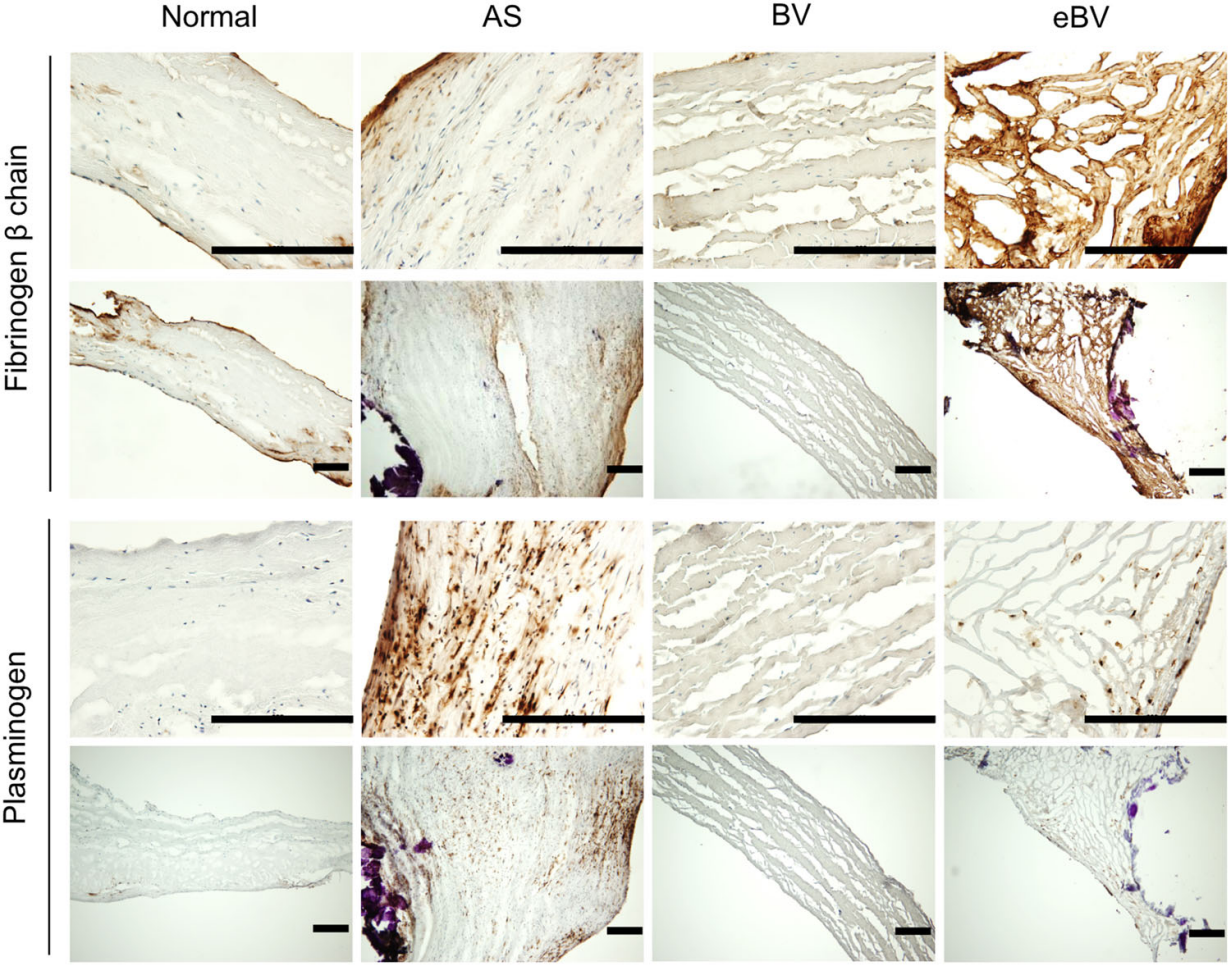
**Immunohistochemistry of fibrinogen beta chain and plasminogen in valve leaflets dissected from normal and AS valves, BV and eBV.** Scale bars: 100 μm.

To clarify the tissue distribution of fibrinogen and plasminogen in calcified valve leaflets, we used immunohistochemistry. Interestingly, fibrinogen was deposited throughout eBV tissue leaflets (Fig. 5). The inner connective tissue layers of normal aortic and AS valves and BVs were hardly stained by anti-fibrinogen beta chain antibody, while the endothelium in these valve leaflets was slightly stained (Fig. 5). On the contrary, plasminogen was dominantly localised on the invasive cells in the interstitial areas of eBV leaflets as well as AS valves (Fig. 5). However, it was not detected in normal valves and BVs, and eBV and SA valves without primary antibodies (data not shown). Importantly, these data were consistent with the western blotting patterns shown in Fig. 4C.

**Fig. 6.**
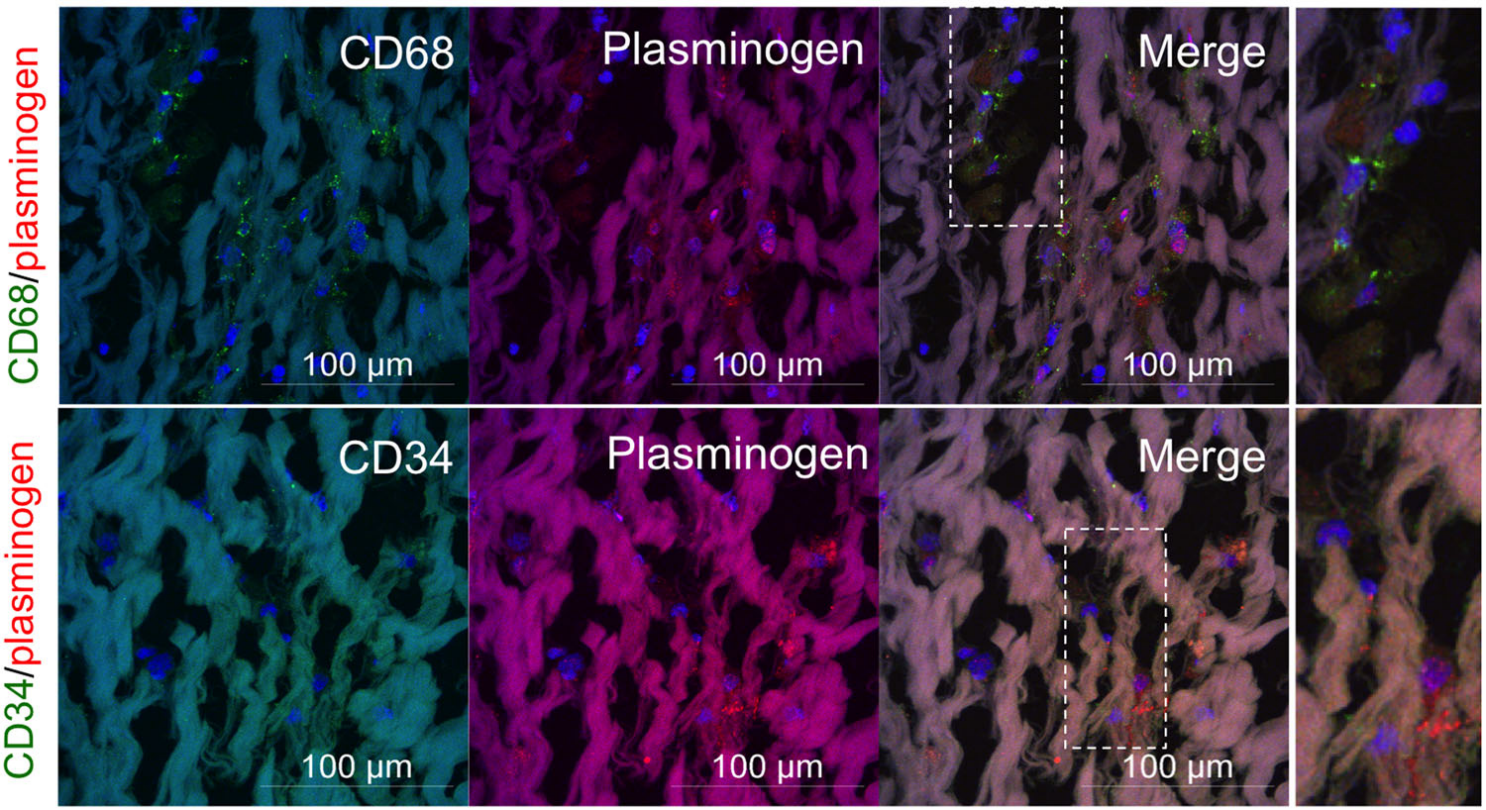
**Immunofluorescence microscopy of frozen sections of eBV.** Valve leaflets were co-stained for anti-CD68/plasminogen or CD34/plasminogen. Nuclei were stained with Hoechst. Scale bars: 100 μm. Right panels are magnified images of the squared areas (broken line) in the merged images.

**Fig. 7.**
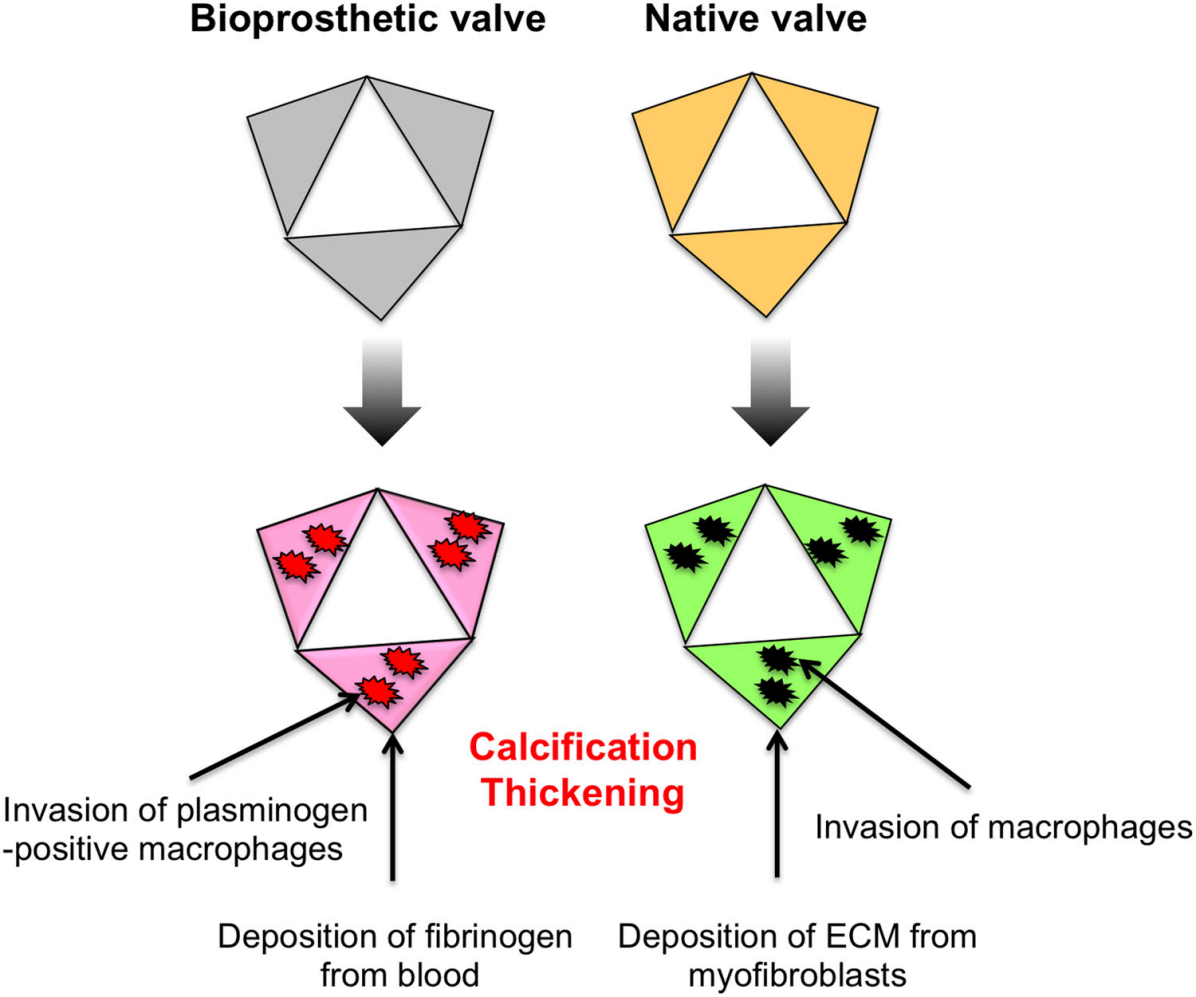
**Schematic illustration of the differential molecular mechanisms of fibrosis and calcification in native valves and explanted BV.** In native valves, when endothelia are damaged, myofibroblasts and macrophages are recruited, resulting in fibrosis and calcification. In BV leaflets, fibrosis occurs through deposition of fibrinogen, and calcification might be directly induced by macrophages.

To identify the cell types expressing plasminogen, we immunofluorescently co-stained plasminogen and cell-type marker proteins, including CD68 (macrophages) and CD34 (fibroblasts). As shown in Fig. 6, Alexa 488 fluorescence from plasminogen was clearly detected in CD68-positive, but not CD34-positive, cells (Fig. 6). In addition, none of the three proteins were detected in BV tissues, and eBV without primary antibodies (data not shown). These data suggested that fibrinogen deposited onto the interstitial extracellular matrix fibre and plasminogen expressed by macrophages might play crucial roles in the degeneration of BV leaflets (Fig. 7).

## DISCUSSION

Recent studies have made extensive progress in revealing the molecular mechanism of calcification in aortic valve leaflets. Normal valvular tissues are maintained by several cell types, including endothelial cells and fibroblasts, and are formed by three main layers, the lamina fibrosa, lamina spongiosa and lamina ventricularis, which are mainly composed of collagen, glycosaminoglycans and elastin, respectively (Lerman et al., 2015; Xing et al., 2004). These connective tissues provide the valve leaflets with the constant strength to resist against the forces of blood flow and pressure. Oxidised low-density lipoprotein is known as an inducer of AS through injuring valvular endothelial cells (Cote et al., 2008; Doherty et al., 2004; Freeman and Otto, 2005; Galle et al., 2006). When endothelial cells are damaged, bone marrow-derived immune cells, such as monocytes and lymphocytes, are recruited into the inflamed valve tissues where they release cytokines such as transforming growth factor-β1 and tumour necrosis factor α. Fibroblasts stimulated by the immune response-mediating factors differentiate into myofibroblasts, which produce extracellular matrix, resulting in fibrosis. At the same time, pre-existing myofibroblasts are expanded. Furthermore, many studies have demonstrated that myofibroblasts continuously exposed to cytokines change into osteoblasts, and this change is involved in calcium-phosphate crystal formation (Mohler et al., 2001; Rajamannan et al., 2003; Yener et al., 2002).

This study provided the first evidence that severe calcification and fibrosis develop in eBVs without myofibroblast recruitment. Indeed, considering the western blotting and immunostaining results, αSMA protein in most AS cases was highly expressed when compared to normal valves. However, αSMA was not detected in eBVs. On the contrary, macrophages were localised around the calcified tissue of eBVs as well as in the tissues of AS valves. This finding suggested that the molecular signal pathways of calcification and fibrosis in BVs might be different from those in AS.

Several recent studies have demonstrated that macrophages contribute directly to tissue calcification in the cardiovascular system (Jeziorska et al., 1998; Li et al., 2016; New et al., 2013). Calcium phosphate crystals are formed on dead macrophages, which are involved in atherosclerotic plaque development, resulting in the calcification of the valvular tissues of eBVs. Interestingly, New et al. (2013) have shown that macrophage-derived matrix vesicles enriched in S100A9 and Anx5 produced micro-calcification in chronic renal disease. Notably, they also showed that few αSMA-positive cells are present in calcified vesicular structures adjacent to CD68-positive macrophages in human plaques. On the basis of this evidence and our findings, we speculate that macrophages might be directly involved in BV calcification, without the differentiation and recruitment of myofibroblasts and osteoblasts.

We next explored the key molecules expressed specifically and dominantly in eBV. Using proteomics techniques, we successfully identified fibrinogen and plasminogen. These proteins are well known as central players of the congealing fibrinogenolysis cascade (Davie et al., 1991). Fibrinogen is cleaved by thrombin, which is involved in fibrin clot formation. In the present study, we found that fibrinogen was distributed throughout the valve tissues. Recently, clinical evidences of the relationship between leaflet immobility and valve thrombosis have been reported. De Marchena et al. (2015) reported layered fibrin thrombus on the aortic side of explanted trans-catheter aortic valve days to months after implantation in several patients and concluded that antithrombotic therapy might be effective for valve thrombosis post-valve replacements (De Marchena et al., 2015). Makkar et al. (2015) reported that thrombosis was seen several years after BV replacement in aortic position, although the ACC/AHA and European Society of Cardiology guidelines do not recommend long-term use of oral anticoagulation with vitamin K antagonists. Mérie et al. (2012) noted that discontinuation of administration of anti-coagulation drug within 6 months after bioprosthetic AVR surgery was related to increased cardiovascular death. These reports suggest that fibrinogen deposited onto BVs might contribute to the formation of fibrin thrombus, resulting in impaired mobility of the valve leaflets. Therefore, additional experiments will be essential to develop novel BV leaflets to avoid fibrin thrombus.

During fibrinolysis, urokinase and streptokinase convert plasminogen to plasmin, which degrades fibrin fibre (Alkjaersig et al., 1958). While this protein is mainly produced by hepatocytes and secreted into the plasma (Bohmfalk and Fuller, 1980), it is also synthesised in human monocytes or granulocytes, depending upon the maturation stage (Prokopowicz, 1968). Our western blotting and immunostaining data showed that plasminogen is present in eBVs as well as AS valve leaflets on macrophages invaded into the calcified area. Interestingly, this protein was not detected in normal valves, although macrophages exist in these tissues. These findings suggested that plasminogen from activated macrophages might contribute to the processes of calcification and inflammation of native and implanted BVs. Indeed, recent studies have demonstrated that plasminogen deposited onto wound tissue positively regulates inflammations by inducing cytokines and intracellular signalling events and potentiating the early inflammatory response (Shen et al., 2012). Furthermore, ovalbumin-induced pulmonary inflammation is strongly inhibited by genetic depletion of plasminogen via suppressing the downregulation of IL-5, tumour necrosis factor-α and gelatinases, which are mediators of asthma and collagen deposition (Swaisgood et al., 2007). Drew et al. (2000) showed that cuff-induced inflammation and neointima formation in model mice of atherosclerotic arteries were completely inhibited by genetic plasminogen depletion. This evidence suggests that plasminogen might be a key factor in tissue inflammation and might be a target for drug treatment of calcification and inflammation of native and implanted BVs. Further investigation using macrophage-specific plasminogen knockout mice are necessary to clarify the roles of the two coagulation-related proteins in calcified and inflamed valve tissues *in vivo*. More importantly, we also need to collect the multiple explanted tissue valves, and quantitative and statistical analysis of the plasminogen and fibrinogen levels will be essential for validation of this study. These clinical experiments would further strengthen our present conclusions. We expect that our data will help to develop novel efficacious therapies for AS.

## MATERIALS AND METHODS

### Patient selection and sample collection

Patients with AS were eligible for this study and were treated according to the American Heart Association (AHA)/American College of Cardiology (ACC) guidelines for the management of patients with valvular heart disease (Nishimura et al., 2017). Aortic Vmax ≥4 m/s or mean pressure gradient ≥40 mmHg was considered severe AS (Nishimura et al., 2017). Explanted valve leaflets were immediately cut into three equal parts with minor axis that were frozen and sectioned for immunohistochemistry or used for protein extraction for western blotting. Histologically and clinically normal aortic valves were taken from necropsied patients within 12 h of death as control samples after formal consent had been given. All experiments were conducted with approval from the institutional ethical committee (Ehime University approved IRB protocol number: 1509022 and 1603002), and written informed consent was obtained from the patients prior to data or sample collection. Patient characteristics are presented in Table 2.

**Table 2.**
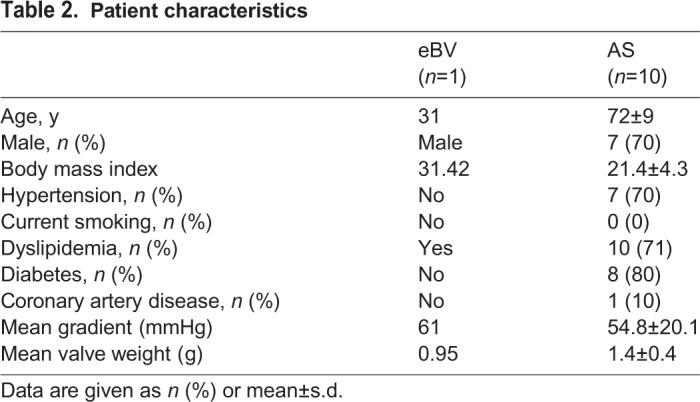
**Patient characteristics**

### Reagents and antibodies

Anti-CD31 rabbit polyclonal antibody was obtained from Spring Biosciences (Catalogue number E11114; Pleasanton, CA, USA). Anti-αSMA antibody was purchased from Novus Biologicals (Clone SPM332; Littleton, CO, USA). Anti-CD68 (Clone PG-M1) and anti-CD34 (Clone QBEnd10) antibodies were obtained from DAKO (Carpinteria, CA, USA). Anti-fibrinogen β-chain and anti-plasminogen antibodies were from Sigma-Aldrich. Alexa Fluor 488-conjugated goat anti-mouse IgG antibody and Alexa Fluor 567-conjugated goat anti-rabbit IgG antibody were purchased from Life Technologies Japan. Bio-Safe Coomassie Blue was purchased from Bio-Rad Laboratories.

### SDS-PAGE

Twenty micrograms of proteins extracted from valve leaflets by homogenisation with Laemmli SDS sample buffer were purified using the methanol–chloroform–water method (Wessel and Flugge, 1984), and then subjected to PAGE (Fig. 4A). Proteins were stained with Bio-Safe Coomassie Blue.

### Western blot analysis

Proteins transferred to Immun-Blot PVDF Membranes (Bio-Rad) were reacted with anti-fibrinogen β-chain, anti-plasminogen, and anti-αSMA primary antibodies. The proteins were visualised using an ECL Prime Western Blotting Detection System (GE Healthcare, Buckinghamshire, UK). Images were taken using an LAS-4000 luminescent image analyser (Fujifilm Life Science Stamford, CT, USA).

### MALDI-TOF/TOF-MS

Proteins were identified as previously described (Sakaue et al., 2017). Briefly, protein bands were trypsinised and analysed using a MALDI-TOF/TOF mass spectrometer (AXIMA-TOF2; Shimadzu, Kyoto, Japan). Spectra were searched using the Mascot search engine (Matrix Science) and against SwissProt 2016_06 (551,385 sequences; https://web.expasy.org/docs/swiss-prot_guideline.html).

### Histological analysis

Cryostat-frozen sections (10 μm thick) from valve tissues were fixed in acetone (4°C, 5 min). For horseradish peroxidase-diaminobenzidine immunostaining, the tissues were processed as described previously (Sakaue et al., 2010). For immunofluorescence staining, after the sections were incubated with primary antibodies (1:100 dilution), Alexa fluorophore-conjugated secondary antibodies were probed, followed by staining with Hoechst. Fluorescent signals were detected using a confocal laser microscope A1R (Nikon Corp, Tokyo, Japan). Haematoxylin and Eosin (H&E) and Masson's Trichrome staining were carried out as described previously (Sakaue et al., 2017). To assess calcium deposition on valve tissues, Von Kossa staining was used. After sectioned specimens were stained with 5% silver nitrate in distilled water for 2 h under exposure to ultraviolet light, calcium oxalate crystals were produced by treatment with 5% sodium thiosulfate for 3 min. For visualisation of glycosaminoglycan, the sections were stained with Alcian Blue as follows. Frozen sections were directly fixed with ethanol and acetic acid and then stained with 5% Alcian Blue solution (pH 2.5). After washing with 3% acetic acid solution, the sections were covered with a cover glass. All images of stained tissues were taken under a phase-contrast microscope (BX51; Olympus, Tokyo, Japan).
